# Intraoperative Safety and Postoperative Complications After SMILE Pro: A Retrospective Case Series of 916 Eyes

**DOI:** 10.3390/jcm15124585

**Published:** 2026-06-12

**Authors:** David Beckers, Florian Kretz, Lena Beckers, Amr Saad, Karsten Klabe, Hakan Kaymak, Mücella Kirca, Detlev Breyer

**Affiliations:** 1 Precise Vision Augenärzte, Osnabrücker Strasse 233, 48429 Rheine, Germany; 2 Institution for International Innovative Ophthalmic Surgery, 40212 Düsseldorf, Germany; 3 Department of Ophthalmology, Stadtspital Zurich, 8063 Zurich, Switzerland; 4 Spross Research Institute, 8063 Zurich, Switzerland; 5 Breyer Kaymak Klabe Augenchirurgie, 40212 Düsseldorf, Germany

**Keywords:** small-incision lenticule extraction, SMILE Pro, femtosecond laser, VisuMax 800, refractive surgery

## Abstract

**Purpose**: To report intraoperative safety and postoperative complications after small-incision lenticule extraction using the 2 MHz femtosecond platform (SMILE Pro; VisuMax 800) in routine practice. **Methods**: Retrospective consecutive case series at a single center. All planned SMILE Pro procedures were analyzed (916 eyes from 482 patients). Outcomes included completion rate, intraoperative events, postoperative complications stratified as <3 and >3 months, and retreatment rate. **Results**: Baseline age was 32.9 ± 6.9 years; average preoperative refraction was −3.60 ± 1.90/−0.87 ± 0.76 D (sphere/cylinder) with best corrected visual acuity of −0.08 ± 0.07 logMAR. Procedures were completed in 911 of 916 eyes (99.45%). Suction loss occurred in six eyes (0.66%); one was completed after redocking, four were converted (two ICL, two femtosecond LASIK) and one did not receive a second procedure. No failed lenticule separations occurred. Retreatment was performed in 14 eyes (1.54%): 11 re-LASIK, 2 ICL, and 1 cataract extraction. Early postoperative events (<3 months) were mainly superficial punctate keratitis (3.51%) and dry eye (1.32%); beyond 3 months, events remained uncommon (dry eye 1.65%, photopsia/halo/glare 0.88%). No severe or sight-threatening complications were observed. **Conclusions**: SMILE Pro on the VisuMax 800 showed a high completion rate, rare intraoperative disruption, low retreatment, and rare, mostly mild postoperative events. These findings support a favorable early safety profile in routine practice; longer-term follow-up is warranted.

## 1. Introduction

Keratorefractive lenticule extraction (KLeX) is a minimally invasive corneal refractive procedure for the correction of myopia and myopic astigmatism [1] and was recently extended to hyperopic treatments [2]. The technique involves the creation and removal of an intrastromal lenticule through a small incision, thereby preserving the anterior corneal lamellae and avoiding flap creation [3]. Compared with LASIK, KLeX maintains greater corneal biomechanical stability and offers high predictability and stability of refractive outcomes [4,5]. Studies have suggested a lower incidence or faster recovery of postoperative dry eye symptoms after SMILE [5]; however, other prospective randomized trials have reported comparable corneal sensitivity and patient-reported dry eye outcomes between SMILE and LASIK [6].

Because the procedure in this study was performed using the VisuMax 800 femtosecond laser system, the established term small-incision lenticule extraction (SMILE) will be used throughout this report [7].

SMILE has demonstrated a high safety profile and reliable refractive outcomes in numerous studies [8,9]. Intraoperative complications such as suction loss or incomplete lenticule dissection occur rarely and can usually be resolved without long-term visual consequences [10]. Postoperative complications, including mild epithelial alterations, transient inflammatory reactions, or interface debris, are generally self-limiting and respond well to conservative management [11]. Severe or vision-threatening events are very rare when adequate patient selection and surgical technique are ensured.

As laser pulse frequencies increase and treatment times decrease, attention has increasingly shifted from refractive efficacy alone toward procedure safety, patient comfort, workflow efficiency, and management of rare intraoperative events. Although improvements in laser speed theoretically reduce treatment duration and suction exposure, real-world evidence regarding the influence of these technological changes on complication profiles remains limited. Characterizing safety outcomes under routine clinical conditions is therefore important for both surgeons adopting new platforms and patients considering lenticule extraction procedures.

The first generation of femtosecond laser systems used for SMILE, operating at a pulse frequency of 500 kHz, has been extensively validated and provides predictable outcomes with low complication rates [12,13,14]. However, the relatively long lenticule creation time of approximately 20 to 25 s required prolonged suction and occasionally led to vacuum loss or patient movement [15]. Furthermore, the available centration control was limited, potentially contributing to minor decentration in some cases [16].

With the VisuMax 800 (Carl Zeiss Meditec AG, Jena, Germany), a 2 MHz femtosecond platform, lenticule creation is executed at a markedly higher pulse-repetition rate, shortening laser-on time and suction duration. By reducing the interval during which fixation must be maintained, the system may lessen intraoperative instability related to eye or head movement. Concurrent refinements in beam delivery, optics, and actuator control are intended to yield cleaner tissue planes and more consistent stromal dissection, thereby improving procedural efficiency and reproducibility [17]. Notably, a shorter and more stable procedure may theoretically reduce mechanical and inflammatory ocular surface irritation, which could contribute to fewer postoperative symptoms. Early clinical data indicate that procedures performed with the 2 MHz laser—commonly referred to as SMILE Pro—achieve comparable or superior refractive outcomes compared with the 800 kHz system, with a similarly low rate of complications [18,19].

Although several recent studies have reported refractive and visual outcomes following SMILE Pro using the VisuMax 800 platform, most have focused primarily on efficacy, predictability, and early visual recovery. Data specifically addressing intraoperative complications, postoperative adverse events, conversion strategies following suction loss, and retreatment patterns remain comparatively limited, particularly in large consecutive real-world cohorts. The present study was therefore designed to evaluate the safety profile of SMILE Pro in routine clinical practice, with a specific focus on intraoperative events, postoperative complications, and retreatment rates in a large series of 916 eyes.

The present study was designed to evaluate the safety and complication profile of SMILE performed with the 2 MHz femtosecond laser system in a large clinical cohort with a follow-up of six months. In this retrospective analysis, intraoperative complications and postoperative symptoms, as well as retreatment rates, were reviewed.

## 2. Methods

### 2.1. Study Design and Patients

This retrospective analysis included all eyes that underwent small incision lenticule extraction (SMILE) using the 2 MHz VisuMax 800 femtosecond laser platform (Carl Zeiss Meditec AG, Jena, Germany) at a single refractive surgery center in Düsseldorf, Germany between January of 2022 and June of 2024. The study adhered to the tenets of the Declaration of Helsinki.

Eligible eyes had undergone SMILE for the correction of myopia or myopic astigmatism with a minimum follow up of six months. Eyes with a history of ocular trauma, corneal disease, previous refractive surgery, or systemic conditions affecting wound healing were excluded. All procedures were performed by one experienced surgeon (D.R.H.B.) following standard protocols for laser setup, docking, and lenticule extraction.

### 2.2. Preoperative Assessment

All patients underwent a comprehensive preoperative examination including manifest and cycloplegic refraction, measurement of uncorrected and corrected distance visual acuity (UDVA and CDVA), slit-lamp biomicroscopy, and fundus evaluation. Corneal topography and tomography were obtained to assess curvature and pachymetry. Patient age and gender were recorded, and only eyes meeting standard safety limits for residual stromal thickness of 250 µm and refractive stability of at least two years were included.

### 2.3. Surgical Technique

The procedure was performed using the VisuMax 800 femtosecond laser with a programmed lenticule diameter of 6.5–7.0 mm, a cap diameter of 7.6 mm, and a cap thickness of 120 to 150 µm. Cap thickness was individualized based on preoperative pachymetry and refractive correction to maintain a minimum residual stromal thickness of 250 µm. A cap thickness of 150 µm was generally preferred and was reduced stepwise toward 120 µm when required. The incision size was set at 2.5 mm with an angle of 135° to facilitate lenticule extraction.

Treatment planning followed a predefined refractive nomogram adopted from the center’s long-standing ReLEx SMILE experience. A systematic 10% additional plus adjustment was applied to the intended spherical correction and this nomogram was used consistently throughout the study period.

During docking, patients were instructed to fixate on the internal fixation target, and centration was achieved on the visual axis.

Following laser application, the lenticule was separated using a blunt dissector and extracted. Postoperatively, all patients received topical antibiotics and corticosteroids for one week, followed by lubricants as needed.

### 2.4. Data Collection and Outcome Measures

All patient records were reviewed to identify intraoperative and postoperative adverse events. Intraoperative complications included suction loss, incomplete lenticule dissection, interface irregularities, or other unexpected surgical events. Postoperative complications were categorized a priori according to their temporal occurrence. Short-term complications were defined as events occurring within the first three months after surgery. Late postoperative events were defined as events arising three months or later postoperatively. Postoperative symptoms (e.g., dry eye symptoms, photic phenomena, diplopia, foreign-body sensation) were extracted from routine clinical documentation and patient-reported complaints recorded during follow-up visits. No validated pre- or postoperative symptom questionnaires were administered as part of standard care during the study period; therefore, symptom frequencies reported herein reflect clinically documented reports rather than standardized patient-reported outcome measures. Retreatment procedures and their indications were recorded separately.

The primary focus of the present analysis was the evaluation of safety-related endpoints, including intraoperative complications, postoperative symptoms, and retreatment rates. In addition, an exploratory vector analysis of astigmatic correction was performed in eyes with available postoperative refractive data. Vector analysis of astigmatic correction was performed in eyes with available 12-month postoperative refractive data. Astigmatic outcomes were evaluated according to the Alpins method using preoperative and postoperative manifest refractive cylinder and axis measurements [20,21]. The Target-Induced Astigmatism (TIA), Surgically Induced Astigmatism (SIA), Difference Vector (DV), Correction Index (CI), Index of Success (IOS), and Angle of Error (AE) were calculated. A CI of 1.0 indicated perfect astigmatic correction, values greater than 1.0 indicated overcorrection, and values less than 1.0 indicated undercorrection. Vector analysis was performed only in eyes with complete refractive follow-up and can be considered an exploratory assessment of refractive efficacy.

### 2.5. Statistical Analysis

All data were compiled and analyzed using Microsoft Excel. Descriptive statistics were used to summarize demographic and clinical data. Continuous variables are presented as mean ± standard deviation, and categorical variables as absolute numbers and percentages.

## 3. Results

All planned SMILE Pro procedures between November 2022 and July 2024 were included in the analysis. We analysed 916 eyes from 482 patients. Of the 482 patients included, 442 underwent bilateral SMILE Pro procedures, while 32 patients were treated unilaterally. The mean age at surgery was 32.9 ± 6.9 years. Preoperative refraction showed a mean manifest spherical error of −3.60 ± 1.90 D, spanning −11.75 to −0.75 D, and manifest cylinder averaged −0.87 ± 0.76 D with a range from −4.75 to 0.00 D. The mean preoperative spherical equivalent was −4.03 ± 1.94 D, with a range from −12.00 to −0.75 D. Visual acuity, recorded clinically in decimal notation, was converted to logMAR for statistical analysis; the preoperative corrected mean was −0.08 ± 0.07 logMAR (range −0.30 to +0.30). Both eyes were included when available (Table 1).

Three-month postoperative refractive data suitable for vector analysis were available for 125 eyes. The mean Target-Induced Astigmatism was 1.05 ± 0.84 D and the mean Surgically Induced Astigmatism was 1.09 ± 0.84 D. The mean Correction Index was 1.10 ± 0.42, indicating a slight tendency toward overcorrection. The mean Difference Vector was 0.26 ± 0.31 D, demonstrating low residual postoperative astigmatism. The mean Index of Success was 0.35 ± 0.51. The mean Angle of Error was −2.7 ± 10.7°, indicating minimal systematic axis misalignment. Within the subgroup with available follow-up data, vector analysis demonstrated good agreement between intended and achieved astigmatic correction (Table 2).

**Figure 1:**
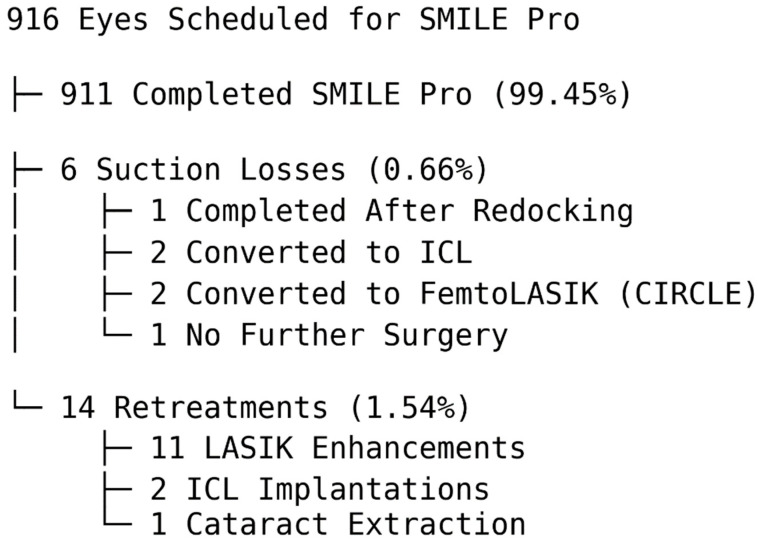
Flowchart of included eyes, intraoperative suction-loss events, conversion procedures, and postoperative retreatments.

911 eyes (99.45%) out of 916 eyes were completed as intended, and 5 eyes (0.55%) were not completed. Intraoperative suction loss occurred in 6 eyes (0.66%). In five eyes (0.55%), suction loss occurred during creation of the posterior (lower) lenticule surface, which precluded continuation of the SMILE procedure and required conversion to alternative techniques. In contrast, in one eye (0.11%), suction loss occurred during creation of the anterior (upper) lenticule surface, allowing successful redocking and completion of the procedure. The five eyes where suction loss occurred in the posterior lenticule surface were managed with alternative strategies—two with posterior chamber phakic IOL implantation, two with femtosecond LASIK conversion using the CIRCLE procedure to convert the SMILE cap into a femtosecond LASIK flap and one with no surgery. No cases of failed lenticule separation were recorded. Refractive retreatments were performed in 14 eyes (1.54%) because of residual refractive error resulting in unsatisfactory uncorrected distance vision, consisting of 11 LASIK enhancements (1.21%), 2 ICL implantations (0.22%), and 1 cataract extraction (0.11%) (Figure 1).

Postoperative events were stratified by time from surgery. Within the first 3 months, superficial punctate keratitis was noted in 32 eyes (3.51%), dry-eye symptoms in 12 (1.32%), photopsia/halo/glare in 5 (0.55%), diplopia in 4 (0.44%), conjunctivitis in 3 (0.33%), foreign-body sensation in 11 (1.21%), and a corneal erosion in 1 (0.11%).

Beyond 3 months, superficial punctate keratitis was documented in 7 eyes (0.77%), dry-eye symptoms in 15 (1.65%), photopsia/halo/glare in 8 (0.88%), diplopia in 4 (0.44%) and conjunctivitis in 4 (0.44%). No cases of persistent foreign-body sensation were recorded in this later interval.

## 4. Discussion

Our series demonstrates a high procedural completion rate with very few intraoperative disruptions and no severe or vision-threatening complications during follow up. Of 916 eyes, 99.45% were completed as intended; suction loss occurred in 0.66% and only one case could be finished after redocking, while five were electively converted to an alternative procedure. No failed lenticule separations were observed. The enhancement rate was low (1.54%), with the majority of cases managed successfully using LASIK enhancement. This rate is lower compared to previously reported enhancement frequencies after SMILE using the VisuMax 500, which range between 2.7% and 4.39% in large series [22,23,24]. The low retreatment rate suggests favorable refractive stability under routine clinical conditions using the VisuMax 800. Early postoperative events were mostly mild and self-limited, while events beyond three months remained infrequent. These findings are consistent with previously published data demonstrating that SMILE is associated with a low incidence of sight-threatening complications and predominantly transient surface-related symptoms. Ganesh et al. reported that the majority of early postoperative findings after SMILE, such as mild interface haze or transient dryness, resolved within the first postoperative month without sequelae [25]. Similarly, Dishler et al. observed that late postoperative events after SMILE are uncommon, with minimal incidence of late inflammatory reactions [26]. Together, these data reinforce the favorable safety profile of SMILE and its low postoperative morbidity, with no severe or sight-threatening adverse events observed in this cohort.

When considered in the context of earlier SMILE experience with the VisuMax 500 platform, the rates of suction loss and postoperative adverse events observed in the present cohort appear numerically lower than those reported in several historical series. However, direct comparisons should be interpreted cautiously because of differences in study design, patient selection, follow-up duration, surgeon experience, and complication definitions. Consequently, the present study was not designed to demonstrate superiority of one platform over another but rather to describe the safety profile of SMILE Pro under routine clinical conditions.

Suction loss remains one of the defining intraoperative complications of lenticule extraction procedures because successful completion depends on uninterrupted laser delivery. In the present cohort, suction loss occurred infrequently and was managed using several different strategies depending on the treatment stage at which suction interruption occurred. The availability of alternative treatment pathways, including redocking, conversion to FemtoLASIK using the CIRCLE procedure, or implantation of a phakic intraocular lens, allowed individualized management without vision-threatening sequelae. These findings highlight that although suction loss remains a relevant intraoperative event, contemporary treatment algorithms provide multiple options for safe and effective management when procedure completion is not feasible.

A series of SMILE performed with the Visumax 500 reported vacuum loss in up to 6.38%, together with incision tears (~9.57%) and epithelial defects (~5.32%), among other issues [15]. Likewise, a large long-term study of 6373 eyes found corneal postoperative complications in approximately 6.8% within 1 year, including punctate epithelial erosions (3.26%) and interface inflammatory phenomena [27]. Although direct comparison is limited by differences in follow-up duration and grading, our suction-loss rate of 0.66% and the low frequency of interface-related findings may be consistent with the technical advantages of newer platforms, which shorten lenticule creation and likely reduce the window for movement and interface disturbance.

Compared with contemporary reports on the VisuMax 800 platform, our findings are broadly consistent with the emerging safety profile of SMILE Pro in routine clinical practice. Saad et al. reported outcomes from 152 eyes at three months, noting no intra- or postoperative complications and no loss of more than one line of corrected distance visual acuity [28]. In a larger series comprising 765 eyes, Cung et al. likewise demonstrated excellent predictability and safety, with a single suction-loss event involving a lenticule remnant (0.13%) and no other intra- or postoperative complications over three months [29]. Our suction-loss rate of 0.66% is marginally higher than that single-center figure yet remains low in absolute terms. Complementary findings were reported by Yoo et al., who compared procedures performed with the VisuMax 800 and VisuMax 500 in a matched case–control design and observed significantly shorter suction times, faster early visual recovery, and no increase in intra- or postoperative adverse events [16]. Similarly, Kim and Chung confirmed these trends in a recent comparative study, demonstrating comparable refractive accuracy and safety between platforms but reduced suction-loss frequency and epithelial disturbance with the 2 MHz system [17]. The enhancement rate observed in our cohort (1.54%) is consistent with previously reported low retreatment rates for the 2 MHz platform and further supports its favorable safety and stability profile under routine clinical conditions [22,30].

This study has several limitations. First, its retrospective observational design and single-center setting limit causal inference and may be subject to selection and reporting biases inherent to real-world case series. In addition, all procedures were performed by a highly experienced refractive surgeon, which may limit the generalizability of the observed complication and retreatment rates to centers with different patient populations, surgical volumes, or levels of surgeon experience.

Second, although this study represents one of the larger safety-focused series of SMILE Pro procedures reported to date, the incidence of key adverse events was very low. Consequently, the present analysis was primarily descriptive in nature and was not designed to identify independent risk factors for rare events such as suction loss, postoperative complications, or retreatment.

Third, follow-up was not specifically designed to capture very late postoperative events. While all included eyes had a minimum follow-up of six months, delayed complications occurring beyond the observation period may not have been detected. Therefore, the present findings should be interpreted as reflecting early and intermediate-term safety rather than definitive long-term outcomes.

Furthermore, symptom-based endpoints such as dry eye, dysphotopsia, diplopia, and foreign-body sensation were derived from routine clinical documentation rather than validated patient-reported outcome instruments. As a result, the reported frequencies likely underestimate the true incidence and severity of subjective postoperative symptoms, limit direct comparison with questionnaire-based studies, and preclude a more detailed assessment of quality of vision and patient satisfaction.

An additional limitation relates to the retrospective collection of adverse-event data. Although all patients underwent standardized postoperative follow-up within routine clinical care, the frequency and detail of symptom documentation may vary between visits and individual patients. Consequently, minor or transient symptoms that resolved spontaneously between scheduled visits may not have been captured. Furthermore, because the study was conducted in a high-volume refractive surgery center with established protocols for patient selection and postoperative management, outcomes may not be fully generalizable to centers with different workflows or patient demographics.

An important strength of the present study is its real-world design. Unlike selected prospective cohorts, the study reflects routine clinical practice, including consecutive patients with a broad range of refractive errors and varying degrees of astigmatism. Such datasets may provide a more representative estimate of complication and retreatment rates encountered in everyday refractive surgery. Although real-world studies inherently sacrifice some degree of standardization, they complement controlled clinical investigations by evaluating the performance of a procedure under conditions that more closely resemble routine patient care.

Another strength of the present study is the consecutive inclusion of all planned SMILE Pro procedures performed during the study period. This approach minimizes selection bias and ensures that uncommon but clinically relevant events, such as suction loss, procedure conversion, and retreatment, are captured within the analysis. In contrast to studies primarily focused on refractive outcomes, the present investigation specifically evaluated the full spectrum of intraoperative and postoperative safety events encountered in routine clinical practice. Such comprehensive event reporting may provide surgeons with a more realistic estimate of the safety profile of SMILE Pro during everyday use. Furthermore, the inclusion of a large number of eyes increases the likelihood of detecting uncommon adverse events that may not become apparent in smaller cohorts.

Because the primary objective of this study was descriptive reporting of safety events rather than formal hypothesis testing, both eyes were included when available. Nevertheless, inclusion of fellow eyes introduces within-subject correlation that is not accounted for in simple descriptive analyses and may slightly influence variance estimates and reported frequencies. Finally, surgeon experience, patient selection, and workflow-related factors may have influenced the occurrence of early intraoperative events such as suction loss and cannot be fully adjusted for within the constraints of this retrospective dataset.

This retrospective study adhered to the tenets of the Declaration of Helsinki. According to local regulations, formal ethics committee approval was not required for retrospective analyses of de-identified data, and the need for individual informed consent was waived.

The study has been evaluated by the Precise Vision Ethics Committee and deemed not to require ethics approval.

No specific funding was received for this study. The authors conducted the research independently without financial support from industry or external organizations.

## Figures and Tables

**Table 1:** Baseline demographic and preoperative characteristics of the study cohort undergoing SMILE Pro (VisuMax 800).

Characteristic	Value
*Eyes (n)*	916
*Patients (n)*	482
Bilateral procedures, *n* (%)	442 (91.7%)
Unilateral procedures, *n* (%)	32 (6.6%)
Mean age at surgery, years	32.9 ± 6.9
Preoperative Manifest Refraction	
Sphere (D)	−3.60 ± 1.90 (range: −11.75 to −0.75)
Cylinder (D)	−0.87 ± 0.76 (range: −4.75 to 0.00)
Spherical equivalent (D)	−4.03 ± 1.94 (range: −12.00 to −0.75)
CDVA (logMAR)	−0.08 ± 0.07 (range: −0.30 to +0.30)

Values are presented as mean ± standard deviation unless otherwise indicated. CDVA = corrected distance visual acuity; D = diopters; logMAR = logarithm of the minimum angle of resolution.

**Table 2:** Alpins vector analysis of astigmatic correction at 12 months postoperatively (*n* = 125). Values are presented as mean ± standard deviation.

Parameter	Mean ± SD
Target-Induced Astigmatism (TIA)	1.05 ± 0.84 D
Surgically Induced Astigmatism (SIA)	1.09 ± 0.84 D
Difference Vector (DV)	0.26 ± 0.31 D
Correction Index (CI)	1.10 ± 0.42
Index of Success (IOS)	0.35 ± 0.51
Angle of Error (AE)	−2.7 ± 10.7°

TIA = Target-Induced Astigmatism; SIA = Surgically Induced Astigmatism; DV = Difference Vector; CI = Correction Index; IOS = Index of Success; AE = Angle of Error.

## Data Availability

The data presented in this study are available from the corresponding author upon reasonable request. The data are not publicly available due to privacy restrictions.
